# Elastocaloric Properties of Polycrystalline Samples of NiMnGaCu Ferromagnetic Shape Memory Alloy under Compression: Effect of Improvement of Thermoelastic Martensitic Transformation

**DOI:** 10.3390/ma15207123

**Published:** 2022-10-13

**Authors:** Francesca Villa, Emanuele Bestetti, Roberto Frigerio, Michele Caimi, Corrado Tomasi, Francesca Passaretti, Elena Villa

**Affiliations:** 1 Consiglio Nazionale delle Ricerche, Istituto di Chimica della Materia Condensata e di Tecnologie per l’Energia (CNR-ICMATE Sede di Lecco), Via G. Previati 1/e, 23900 Lecco, Italy; 2 Dipartimento di Meccanica, Politecnico di Milano, Via La Masa 1, 20156 Milano, Italy; 3 Dipartimento di Scienza dei Materiali, Università di Milano Bicocca, 20125 Milano, Italy; 4 Consiglio Nazionale delle Ricerche, Istituto di Chimica della Materia Condensata e di Tecnologie per l’Energia (CNR-ICMATE Sede di Genova), Area della Ricerca di Genova, Via De Marini 6, 16149 Genova, Italy

**Keywords:** ferromagnetic shape memory alloys, NiMnGaCu, microstructure, mechanical properties, thermal analysis, elastocaloric effect

## Abstract

Shape memory alloys (SMAs) and ferromagnetic shape memory alloys (FeSMAs) have recently attracted interest for solid state refrigeration applications. Among NiMnGa-based quaternary systems, NiMnGaCu exhibits an interesting giant magnetocaloric effect thanks to the overlapping of the temperatures related to the magnetic transition and the thermoelastic martensitic transformation (TMT); in particular, for compositions with Cu content of approximately 6 at%. In the present work, we investigated the improvement effect of TMT on the total entropy change (*ΔS*) in the elastocaloric performances of polycrystalline Ni_50_Mn_18.5_Cu_6.5_Ga_25_ at% alloy samples, just above room temperature. We report an extensive calorimetric and thermomechanical characterization to explore correlations between microstructural properties induced by the selected thermal treatment and elastocaloric response, aiming at providing the basis to develop more efficient materials based on this quaternary system. Both *ΔT* and *ΔS* values obtained from mechanical curves at different temperatures and strain recovery tests under fixed load vs. T were considered. Maximum values of *ΔS* = 55.9 J/KgK and *ΔT* = 4.5 K were attained with, respectively, a stress of 65 MPa and strain of 4%. The evaluation of the coefficient of performance (*COP*) was carried out from a cyclic test.

## 1. Introduction

Heusler-type ferromagnetic shape memory alloys (FeSMAs) based on the NiMnGa system are a class of smart environment-friendly materials that have received increasing attention since the 1990s [1,2,3,4]. Due to their peculiar magneto-mechanical properties, these alloys can find potential applications in the fields of magnetic refrigeration, actuators, sensors and energy harvesters [5,6,7,8,9,10,11,12,13]. In particular, FeSMAs alloys exhibit the so-called giant magnetocaloric effect thanks to the strong coupling between magnetic and elastic degrees of freedom, which can be optimized by tuning the thermoelastic martensitic transition (TMT) through doping with an additive metallic element to overlap with the magnetic transition temperature [14,15,16,17]. Besides their other functional properties, such as elastocaloric effects, we aimed to investigate these alloys from magneto-mechanical coupling perspectives.

In the last few years, a number of investigations have been devoted to studying various properties of doped NiMnGa SMA, both in single crystals and polycrystalline samples, as well as in films and microwires.

The superelastic behaviour and the elastocaloric effect of [001]-textured polycrystalline Ni_50_Mn_23_Ga_25_Cu_2_ were investigated by Guo et al. below room temperature [18]. The authors found a good balance between the adiabatic temperature change *(ΔT_ad_*) and the coefficient of performance (*COP_mat_*), suggesting that this material can be a promising candidate for low-temperature elastocaloric refrigeration. There are only a few papers, in addition to this latter work, focused on the elastocaloric investigation of NiMnGaCu alloy, since this system is principally studied as a magnetocaloric material. Recently, Li et al. [19] reported some promising elastocaloric properties, in terms of adiabatic *ΔT*, for polycrystalline NiMnGaCu samples with high Cu content (20 at%) obtained from directional solidification. Another significant experimental study was carried out by Gràcia Condal et al. [20], who focused their work on the magneto-mechanical coupling in NiMnGaCu through calorimetric measurements under magnetic-field and controlled stress.

Gao et al. [21] published research on the relationship between magnetoresistance and the martensitic transformation (MT) in Ni_48_Mn_22_Ga_22_Co_4_Cu_4_ shape memory alloy microwires. They observed that the microwires’ diameter had an influence on the martensitic transformation that could be explained in terms of different effective cooling rates during the rapid quenching in the wires’ preparation process.

Similarly, Zhang et al. [22] focused their attention on the phase transformation temperature and the elastic properties of Ni_53_Mn_24_Ga_21_CoCu microwires. Using 140 μm wires, the authors reported superelasticity with a giant recoverable strain that remained largely unchanged after a 100 cycle test at 525 K in a wide temperature range.

Pérez-Checa et al. [23] studied the role played by Fe added to the Ni_45_Mn_25_Ga_20_Co_5_Cu_5_ shape memory alloy (SMA) in high temperature actuators. The authors observed that the addition of 5 at% of Fe led to the most promising candidate, with both high TMT and Curie (T_c_) temperatures and a low c/a tetragonal ratio.

An earlier paper from the same group reported a study on the critical parameters responsible for the magnetic field-induced strain in Ni(Co)-Mn(Fe)-Ga(Cu) single crystals at high temperatures [24].

Zelený et al. [25] investigated the influence of Co and Cu doping on magnetocrystalline anisotropy (MCA) in Ni_2_MnGa tetragonal non-modulated martensite. They demonstrated how doping can increase the TMT but, at the same time, reduces the magnetocrystalline anisotropy down to zero for high Co/Cu concentrations.

A study on the microstructural anisotropy and magnetic properties of as-cast and annealed NiMnGaCoCu melt-spun ribbons was published by Brzoza et al. [26]. In the two-step annealing process of the ribbons, the authors first found a rise in the atomic ordering that led to an increase in the magnetic moment and, subsequently, the sample’s transformation into a single non-modulated phase.

Mashirov et al. [27] recently reported the shape-memory effect on Ni_50_Mn_18.5_Ga_25_Cu_6.5_ microsized samples produced by a focussed ion beam. The authors stated that the elastic strain was observed in the microcantilever during the longitudinal-transverse bending in the martensite phase, but a two-sided shape-memory effect was not detected.

Bodnárová et al. [28] compared the elastic tensors of Mn-rich and doped NiMnGaCoCu non-modulated martensite, using both experiments and ab initio calculations based on density functional theory (DFT). They proved that two similar materials undergoing a cubic-to-tetragonal martensitic transition along the same strain path and having similar lattice parameters can exhibit very different elastic properties in the martensite phase.

Thin films of high-temperature NiMnGa FMAs doped with Fe, Co and Cu were investigated by Alexandrakis et al. [29]. Weak dependence on doping for both the martensitic transformation temperature and tetragonality was described. The irregular behaviour of the latter parameter was ascribed to the competitive influence of the doping elements and the fluctuations of the Ni concentration.

In our previous work [30], the correlation between the microstructure and magnetocaloric properties of polycrystalline Ni_50_Mn_18.5_Cu_6.5_Ga_25_ FeSMA, obtained by magnetization measurements under different magnetic fields, was investigated. A complete calorimetric and structural characterization of some selected samples was performed and a new parameter, *CE_f_*, was introduced to express the efficiency of the martensitic transformation:

*CE_f_* = *ΔH*/*ΔT* (1)

This coefficient was obtained from calorimetric analysis, where *ΔH* is the enthalpy involved in the martensitic transition and *ΔT* is the temperature range where the transition happens. This parameter is simply based on experimental data and has no particular physical significance. However, it is able to express in a quick way the efficiency of the thermoelastic transformation. In fact, in this way, it is possible to highlight not only the importance of the value of enthalpy involved in the transformation but also the significance of the corresponding small temperature range of the transition. Indeed, a small *ΔT* is related to a rapid transition, which involves the simultaneous transformation of a large amount of material. More specifically, if the martensitic domains have homogeneous sizes and dimensions, the TMT occurs in a more homogeneous and simultaneous way, since the whole material transforms at the same time in correspondence with a narrow range of temperatures. In this way, it is possible to enhance the microstructure to optimize the thermoelastic martensitic transformation. In the present work, we considered the effect of such a parameter in depth and also extended the analysis to the elastocaloric performances. Hence, our attention was focused on the elastocaloric properties of two polycrystalline Ni_50_Mn_18.5_Cu_6.5_Ga_25_ FeSMAs with different *CE_f_* values obtained in a compression configuration. The results are expressed in terms of *ΔS* evaluated from both strain recovery and stress–strain mechanical tests. Moreover, the coefficient of performance (*COP*) was evaluated for the examined samples. Finally, the role of the microstructure on the TMT efficiency and, in turn, on the total *ΔS* was assessed.

## 2. Experiment

Ni_50_Mn_18.5_Cu_6.5_Ga_25_ (in at%) ingots were prepared in a non-consumable electrode vacuum arc furnace (Leybold LK6/45) from pure (>99%) metallic elements. The ingots were melted into a water-cooled copper crucible under a protective atmosphere (pure 99.999 Ar), and they were re-melted six times to improve the chemical homogeneity. In accordance with our previous study [30], two representative thermal treatments were chosen for this new investigation: TT at 1123 K for 6 h under vacuum conditions followed by slow cooling (≈1 K /min) to room temperature (samples of the “A” series) and TT at 1123 K for 12 h in an inert atmosphere followed by water quenching (samples of the “B” series). The samples obtained after these two thermal routes presented different *CE_f_* coefficients and sufficient mechanical properties, promising for a significant characterization of the elastocaloric properties.

The calorimetric analysis was performed with a Q200 TA Instruments differential scanning calorimeter (DSC), with a liquid nitrogen cooling system in the [223 K; 370 K] temperature range with a rate of 10 K/min and in a He gas atmosphere of 25 mL/min. The characteristic temperature measurements for both martensitic and reverse transitions were determined using the tangent method intersection between the tangent of the base line and the tangent at the peak inflection point (onset point). The enthalpy values were obtained by integration of the complete transition peak.

Optical microscopy (OM) images of mechanically polished samples were taken using a Leitz-ARISTOMET light microscope. Before OM observations, samples were chemically etched with Marble’s reagent for 30 s.

Mechanical tests were performed with an E3000 Instron equipment with a thermal control chamber in compression configuration. The adiabatic *ΔT* measurements were obtained with an ad hoc set-up, with T-type thermocouples controlled in Labview (2020 community edition) software program.

Samples of 3 × 3 × 8 mm^3^ in size were prepared for mechanical analysis in a compression configuration. The mechanical stress–strain curves were registered in stress control at different temperatures with a rate of 10 MPa/min. The strain recovery tests were carried out under constant loads in the [50MPa;200 MPa] range, with a rate of temperature change of 3 K/min. The stress–strain cycle tests for the *COP* measurement were performed at 353 K up to 4% strain. The best adiabatic measures of *ΔT* were registered with a strain rate of 200%/min in the loading stage and 600%/min in the unloading stage, after iso-stress steps of 40 s and up to a strain of 4% and a stress of −120 MPa.

## 3. Results

Following the procedure previously described, two series of NiMnGaCu polycrystalline samples—namely, A and B—were prepared. The samples were checked with DSC analysis to ascertain the different correspondences of the *CE_f_* values, strictly related to the thermoelastic martensitic transformation (TMT) efficiency. The transformation temperatures (in K) related to the A samples were *M_s_* = 321.7, *M_f_* = 313.6, *A_s_* = 323.6 and *A_f_* = 330.4, while the temperatures for the B samples were *M_s_* = 316.7, *M_f_* = 313.4, *A_s_* = 321.5 and *A_f_* = 326.3. Using Equation (1), we obtained an average CE*_f_* value of 0.45 J/gK for the A series (TT at 1123 K for 6 h followed by slow cooling (SC)) and 0.91 J/gK for the B series (TT at 1123 K for 12 h followed by water quenching (WQ)).

**Figure 1:**
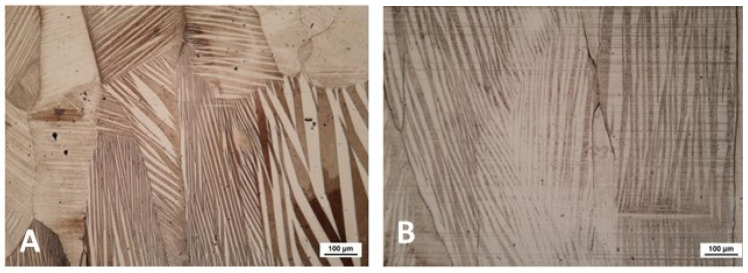
Optical microscopy observations for samples A (**A**) and B (**B**).

A couple of examples of the grain structure, observed by optical microscopy, are reported in Figure 1. The A samples showed complex modulation of the martensitic grain structure, characterized by large grains (in the order of hundreds of μm) oriented in the cooling direction. The B samples showed a similar grain size but a more homogeneous modulation of martensite. Even if the microscopy observation only provided a qualitative analysis of the microstructure, it is reasonable to consider that such a structural aspect contributed to the narrow temperature range of the TMT. In contrast, in the case of the A samples, the formation of twins with very different sizes and modulations could lead to a transition spread smeared over a wider range of temperature. This effect was easily and simply represented by the CE*_f_* coefficient calculated from the DSC analysis.

Strain recovery measurements vs. *T* under different stresses were carried out in the [210–480 K] temperature range and in the [50–200 MPa] stress range through compression testing.

**Figure 2:**
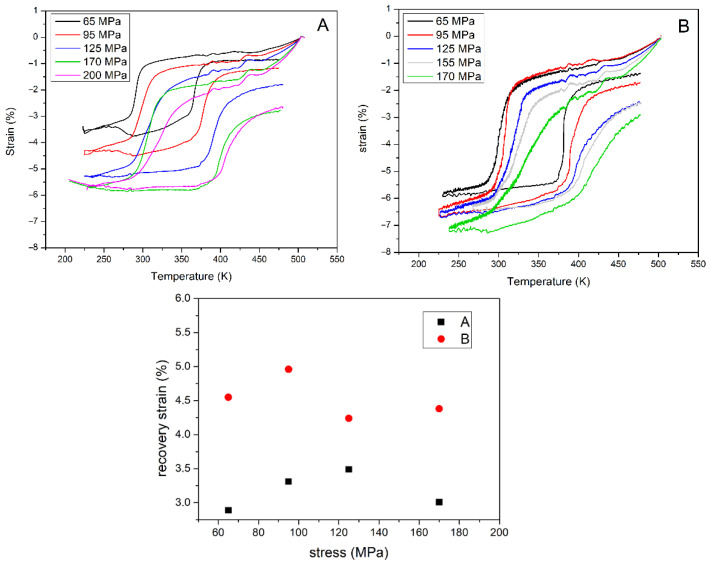
Strain recovery test vs. *T* under fixed stress and recovery strain vs. stress for samples A (**A**) and B (**B**).

In order to collect reliable data, the raw curves were accurately elaborated to subtract the thermal expansion contribution of the compression platens and to insert corrections for thermal deviation from the temperature ramp defined. An accurate calibration process was designed by using different thermocouples to control the correct sample temperature. Aberrations due to thermal expansion of the experimental setup were estimated by recording a baseline on a quartz sample. The elaborated curves of the strain recovery for samples A and B are presented in Figure 2. Concerning the strain recovery properties, it is important to highlight that the two series of samples also reflected the more efficient TMT in the strain recovery properties, even if the residual strain shown at the higher stress applied was correlated with irreversible plastic deformation, which is unavoidable in these kinds of alloys. In the inset of Figure 2, it is possible to see that the B samples had higher values for the recovery strain, with a maximum value of 4.96% obtained for the applied stress of 95 MPa in contrast to the value of 3.31% measured for sample A.

A significant residual strain (up to 2.5–2.7 %) after compression at different loads and the consequent recovery process vs. T was registered for all the analysed samples. The maximum strain recovered for sample A was about 2.5%, while sample B attained values of about 4%.

The efficiency of TMT was represented by both higher strain recovery values and a narrower temperature range corresponding to the main strain induction and recovery, which strictly corresponded with the width of the calorimetric transition peak. One of the first and most important theoretical approaches to the experimental evaluation of elastocaloric properties was reported in reference [31]. In light of this discussion, and considering the Maxwell equation (Equation (2)), it was judged that the efficiency of TMT is also reflected in the calculation of the elastocaloric entropy change (*ΔS*) from these strain recovery measures, where the derivative of strain is an important contribution:

(2) $$\Delta S=\int_{0}^{\sigma }\frac{d\varepsilon \;}{dT}d\sigma$$

At fixed stress, the evaluation of *ΔS* from the discrete integration of the derivative vs. *T* of the strain recovery *ε* curves can be achieved.

**Figure 3:**
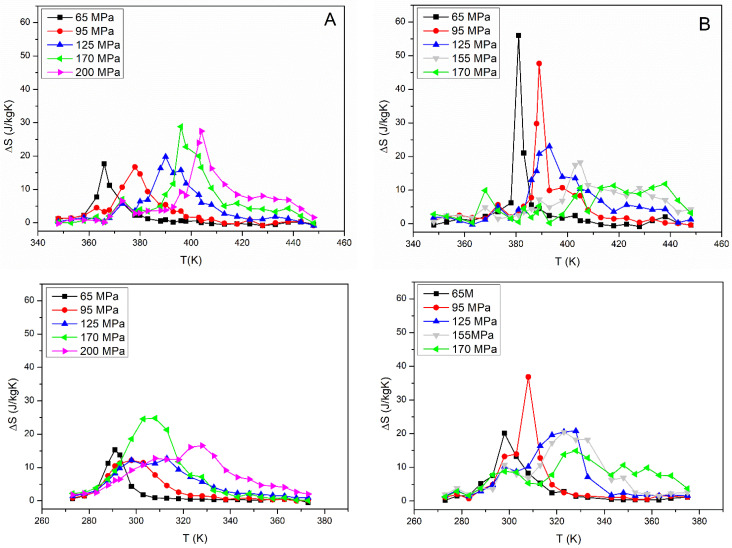
Value for *ΔS* vs. *T* obtained by discrete integration from strain recovery curves in the heating (**top**) and cooling (**bottom**) parts for samples A (**A**) and B (**B**).

In Figure 3, we report the results of the discrete integration of the strain recovery curves in heating and in cooling stages for samples A and B. The maximum obtained value of *ΔS* was 55.9 J/KgK for sample B under a load of 65 MPa. Sample A showed lower values and the best result was 28.8 J/KgK under a load of 95 MPa. Generally, higher values for entropy were registered in correspondence with the heating part of the curves.

A series of stress–strain curves recorded at different temperatures were also considered. Due to the poor mechanical properties of the analysed system (e.g., brittleness), both samples A and B yielded lesser quality (measurements /results) compared to the strain recovery ones.

Although plastic deformation plays a chief role in triggering sample degradation, making comparison between curves collected from different specimens difficult, the data were more than enough to allow an evaluation of the entropy change performance.

Even in this case, by using the Maxwell equation (Equation (3)), the entropy change during the loading and unloading stages [31] could be obtained from discrete integration.

(3) $$\Delta S=-\int_{0}^{\varepsilon }\frac{d\sigma \;}{dT}d\varepsilon$$

**Figure 4:**
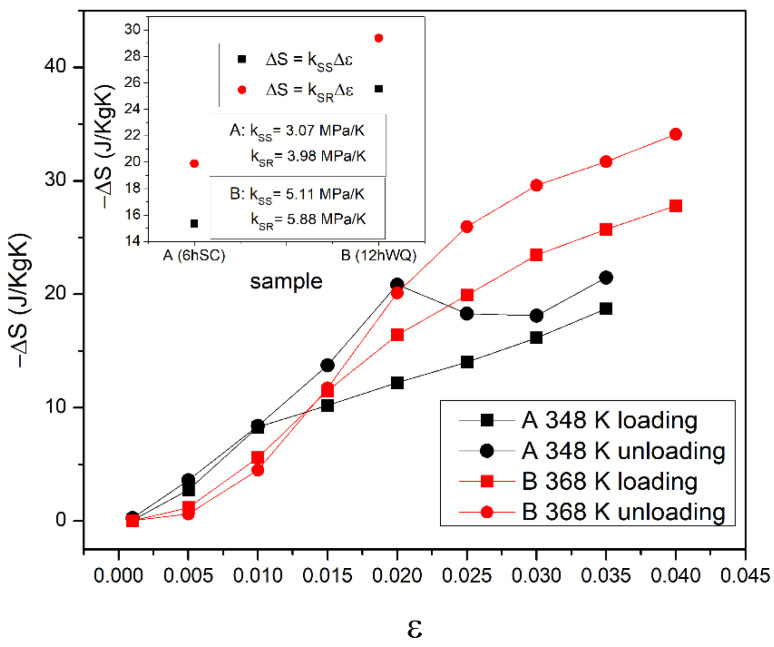
Value for *ΔS* vs. strain obtained using discrete integration from stress–strain curves at different temperatures in the loading and unloading stages for samples A and B.

Figure 4 depicts the *ΔS* vs. strain (*ε*) at different temperatures (*T*).

The inset shows the general estimation of *ΔS* using the Clausius–Clapeyron coefficient *K* = d*σ*/d*T*, following the theoretical evaluation as reported in Equation (4) [31]:

(4) ΔS=−KΔε

where *Δε* is the associated strain of the stress–strain curves at the different curves considered. The *K* values were obtained from the stress–strain curve analysis at different temperatures and from the strain recovery test vs. *T* at different loads, using the correlation for the *A_s_* temperature. The results are also reported in Table 1.

It is worth noting that a density reference value of 8.2 g/cm^3^, taken from the literature, was used for the entropy evaluation of all the analyzed samples.

The values obtained for the *ΔS* were in good agreement with the first evaluation from the strain recovery curves. The maximum value for sample A was 21.48 J/KgK in the unloading phase at 0.035 strain. At the same strain, the value for sample B was 31.7 J/KgK in the unloading phase. Using the Clausius–Clapeyron *K* coefficient, the values were 19.9 J/KgK and 29.4 J/KgK for samples A and B, respectively (see Figure 4). These values can be compared with ≈20 J/KgK and 48–50 J/kgK for samples A and B, respectively, obtained from strain recovery tests under heating, at the same strain.

**Figure 5:**
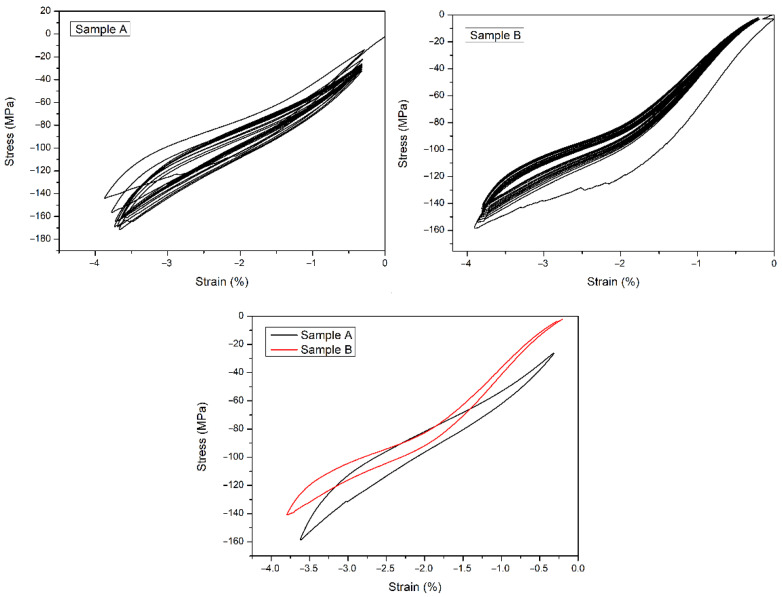
Stress–strain curves at 353 K for *COP* evaluation.

Some of the cycles of the stress–strain curves at 353 K with a strain rate of 1%/min provided further guidance for better understanding the elastocaloric properties of samples A and B, allowing a first indication about the intrinsic *COP*. As reported in Figure 5, the values of the *COP* were quite similar for the two series of samples: 19 for sample A and 19.7 for sample B. Nevertheless, both the stability and the quality of the pseudoelastic curves were better in sample B. In Figure 5, a useful comparison between the stabilized stress–strain cycles for the two samples A and B is also shown. The evaluation of the intrinsic *COP* can be obtained by using Equation (5):

(5) $$\;COP=\frac{Q}{W}=\frac{mc\Delta T}{\frac{1}{\rho }\oint \sigma d\varepsilon }$$

Finally, in order to consider the experimental evaluation of *ΔT*, let us take into account Equation (6), which leads to a first intrinsic value given by the theoretical approach from calorimetric analysis:

(6) $$\Delta T=\frac{\Delta H}{c_{p}}$$

where *ΔH* is the enthalpy variation and *c_p_* is the specific heat measured in the calorimetric analysis. Using a reference *c_p_* determined in our previous work [30], quite high *ΔT* intrinsic values of 20.85 K and 24.5 K for samples A and B, respectively, were obtained.

**Figure 6:**
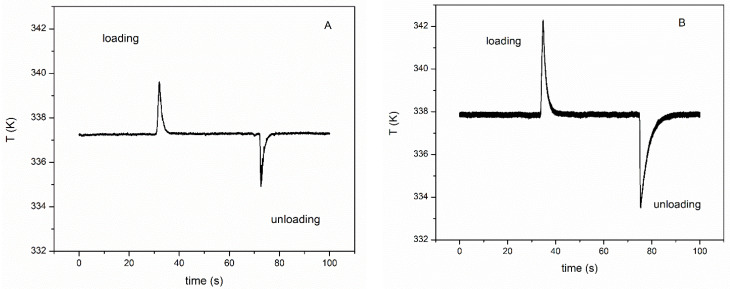
*ΔT* range for samples A (**A**) and B (**B**) measured by ad hoc prepared thermocouples system.

The experimental *ΔT* values measured from an optimized system of thermocouples are displayed in Figure 6, where a useful comparison between the *ΔT* curves of samples A and B is reported. The *ΔT* values were higher for sample B than those for sample A, with average values of 4.5 K and 2.37 K, respectively. It is also interesting to note that values registered in the unloading part of the curve were generally higher, by about 10%, than those recorded in the loading part.

## 4. Discussion

In this work, the foremost aim was to organize a complete experimental investigation to highlight the influence of optimized TMT on elastocaloric properties. It is not wrong to consider that, even if this NiMnGaCu alloy shows the problem of brittleness usually found in FeSMA alloys, it might be possible to achieve acceptable mechanical properties in compression configuration, thus allowing an initial characterization of elastocaloric properties. Moreover, the optimization of the grain structure through thermal treatments and the consequent improvement of the TMT efficiency enhance the elastocaloric parameters *ΔS* and *ΔT*. The correlation between the *CE_f_* parameter and the elastocaloric response was confirmed by different kind of measurements.

In Table 2, we summarize the principal peak parameters registered in our work to provide a complete comparison between the different experimental procedures.

Despite this point of view, the same hint is apparent; i.e., sample B, with higher *CE_f_* and more efficient TMT, showed the highest values for both *ΔS* the from mechanical curves (55.9 J/kgK) and *ΔT* (4.5 K).

It is interesting to underscore that the mechanical test of the strain recovery led to a larger deformation and more precise measurement repeatability, even in the case of indirect, long and expensive measurements. In the stress–strain configuration, the stronger influence of the plastic deformation, as well as the irreversible break of the sample, limit the investigation to a few curves at reduced levels of strain. This is the principal reason why the *ΔS* values registered in the stress–strain curves were more underestimated than those recorded from the strain recovery curves, particularly for the B samples. Furthermore, the results obtained from the stress–strain curves were less coherent: for example, the values obtained for sample A did not completely increase with the strain as expected. It is possible that, during the unloading stage, some effect from the plasticity or induction of defects occurred, lowering the stress values and the correspondent entropy change. It is also interesting to consider that, in the mechanical characterization, the *ΔS* was related not only to the thermally induced TMT but also to the contributions of the different degrees of order between twinned and detwinned martensite and, finally, the elastic deformation part. Finally, from the numerical point of view, in the *ΔS* calculated from the Maxwell equations, a huge contribution was provided by the strain derivative vs. temperature. In these NiMnGaCu samples, which present high efficiency in the TMT, the slope of the strain curves in correspondence with the transformation was quite high and, therefore, the derivatives also reached significant values. In general, it is possible to observe that the entropy change is affected by thermal and structural factors and the values evaluated in different test situations could consequently show some differences.

The first evaluation of the *COP* started by considering the theoretical approach described in Equation (5). In this case, only the intrinsic contributions of heat released and mechanical work were used. There was no evaluation of the real thermodynamic efficiency in the one cycle where heat transfer was included. However, it was a first indication that can give some information about microstructure optimization, and it was not so far from other values obtained in the literature [18]. The tuning of the ratio between the heat involved and the mechanical work associated with the mechanical hysteresis is the principal way of obtaining a more promising material from the elastocaloric point of view.

The difference between the intrinsic elastocaloric potential and its expression and evaluation in practical applications is clearly apparent when comparing the *ΔT* values calculated from enthalpy and calorimetric measurements with those experimentally obtained from mechanical curves, where the total heat transfer associated with the full stress-induced martensitic transformation was affected by structural defects and energy dispersion due to the actual microstructure. Moreover, the adiabatic *ΔT* was strongly influenced by the geometry of the samples and the heat transmission conditions. Indeed, in the current case, the samples were characterized by a low surface/volume ratio and they worked between the two compression platens, and this affected the adiabatic *ΔT* direct measurements. Moreover, the theoretical *ΔT* obtained from the calorimetric measurement was significantly higher and it could be considered as an intrinsic maximum value for the NiMnGaCu material. However, both the theoretical and measured adiabatic *ΔT* were coherent with the microstructural condition of the samples, since the B sample, with a better optimized microstructure, presented higher *ΔT* values.

Generally, the trend in the experimental parameters was confirmed in any case. Furthermore, it was experimentally demonstrated that a TMT occurring in a narrow temperature range and in a homogeneous way inside the material was reflected in improved elastocaloric performances.

It is also worth noting that the best results on this alloy were achieved by the reverse transformation; i.e., in the heating part of the strain recovery curves and in the unloading stage of the stress–strain mechanical curves, just as in the case of the magnetocaloric *ΔS* study reported by Sarkar et al. [32].

In the literature, there are a few results from experimental measurements of *ΔS* values for NiMnGaCu, particularly from analyses of strain recovery curves and compression tests with bulk samples. Nevertheless, the *ΔS* values reported in [20] are in good agreement with these results and they are able to demonstrate the effect of the performance improvement due to TMT optimization. Considering the adiabatic *ΔT*, Li et al. [19] directly measured higher values, but they studied NiMnGaCu samples with higher Cu content, which could improve the mechanical response of the alloy.

The tuning of microstructures could be an important route to improve functional properties and investigate the exact correlation between intrinsic material parameters and solid state refrigeration properties. It is crucial also to collect important information about the challenges of using and exploiting magneto-mechanical coupling in these kinds of systems.

## 5. Conclusions

Two kinds of NiMnGaCu polycrystalline samples with different thermal process routes of preparation were examined with a mechanical experimental test to evaluate elastocaloric parameters from different points of view. Therefore, B sample with a more efficient *CE_f_* coefficient and, consequently with more efficient TMT showed the best performance, with a maximum *ΔS* of 55.9 J/KgK registered in the strain recovery test and of 34.1 J/KgK in the stress–strain test. The maximum *ΔT* measured for these samples was 4.5 K. The correlation for the microstructure corresponding to more efficient TMT and functional properties was experimentally investigated. Based on the first indications obtained from the optical microstructure, the key material parameters seemed to be the more homogeneous modulation of martensite in the B samples, given that the samples showed similar average grain sizes. Further, the better mechanical performances were related to the quicker and easier induction process for martensite from stress. This indication will be further investigated using diffraction analysis. Even if the poor fatigue properties of this alloy hinder the development of devices based on it, this extensive characterization study provides important information about the development of multifunctional materials for solid state refrigeration based on NiMnGaCu alloy compositions with the possibility of magneto-mechanical coupling.

## Figures and Tables

**Table 1:** Registered values for Clausius–Clapeyron coefficient *K* for samples A and B.

Sample	*K* from Strain Recovery Test	*K* from Stress-Strain Test
A (6 h SC)	3.98	3.07
B (12 h WQ)	5.88	5.11

**Table 2:** Peak values and maximum values for enthalpy change *ΔS* and *ΔT* for the A and B sample series. The *ΔS* values are expressed in J/kgK. CC/SS and CC/SR are the entropy change values obtained using the Clausius–Clapeyron coefficient obtained from stress–strain (SS) curves or strain recovery (SR) curves; the *ΔT* values are expressed in K.

Sample	*ΔS* (SR Cooling)	*ΔS* (SR Heating)	*ΔS* (SS Loading)	*ΔS* (SS Unloading)	*ΔS* (CC/SS)	*ΔS* (CC/SR)	*ΔT* (DSC)	*ΔT* (exp)
A series	24.9	28.8	18.7	21.4	15.4	19.9	20.85	2.37
B series	36.9	55.9	27.7	34.1	25.6	29.5	24.5	4.5

## Data Availability

The data presented in this study are available on request from the corresponding author.
